# Amorphous InGaMgO Ultraviolet Photo-TFT with Ultrahigh Photosensitivity and Extremely Large Responsivity

**DOI:** 10.3390/ma10020168

**Published:** 2017-02-13

**Authors:** Yiyu Zhang, Ling-Xuan Qian, Zehan Wu, Xingzhao Liu

**Affiliations:** 1 School of Microelectronics and Solid-State Electronics, University of Electronic Science and Technology of China, Chengdu 610054, China; 201211030305@std.uestc.edu.cn (Y.Z.); 201421030407@std.uestc.edu.cn (Z.W.); 2 State Key Laboratory of Electronic Thin Films and Integrated Devices, Chengdu 610054, China

**Keywords:** InGaMgO, post-deposition annealing, ultraviolet photo-TFT, oxygen vacancy, photosensitivity, responsivity

## Abstract

Recently, amorphous InGaZnO ultraviolet photo thin-film transistors have exhibited great potential for application in future display technologies. Nevertheless, the transmittance of amorphous InGaZnO (~80%) is still not high enough, resulting in the relatively large sacrifice of aperture ratio for each sensor pixel. In this work, the ultraviolet photo thin-film transistor based on amorphous InGaMgO, which processes a larger bandgap and higher transmission compared to amorphous InGaZnO, was proposed and investigated. Furthermore, the effects of post-deposition annealing in oxygen on both the material and ultraviolet detection characteristics of amorphous InGaMgO were also comprehensively studied. It was found that oxygen post-deposition annealing can effectively reduce oxygen vacancies, leading to an optimized device performance, including lower dark current, higher sensitivity, and larger responsivity. We attributed it to the combined effect of the reduction in donor states and recombination centers, both of which are related to oxygen vacancies. As a result, the 240-min annealed device exhibited the lowest dark current of 1.7 × 10^−10^ A, the highest photosensitivity of 3.9 × 10^6^, and the largest responsivity of 1.5 × 10^4^ A/W. Therefore, our findings have revealed that amorphous InGaMgO photo thin-film transistors are a very promising alternative for UV detection, especially for application in touch-free interactive displays.

## 1. Introduction

The amorphous oxide semiconductors based thin film transistors (TFTs), represented by amorphous InGaZnO (a-IGZO) TFTs, have been intensively developed for a variety of applications in flat panel displays (FPDs), including electronic papers (e-papers), organic light-emitting-diode displays (OLEDs), and liquid crystal displays (LCDs) [1,2,3]. In addition, a-IGZO TFTs have also been demonstrated to act as ultraviolet (UV) photo thin-film transistors (photo-TFTs) employed in an active matrix, accordingly realizing the contact-free interactive display [4]. For example, Tze-Ching Fung et al. studied both the wavelength and intensity dependent photo-responses in a-IGZO photo-TFTs, suggesting the possibility of using a-IGZO TFT as UV-light photo-sensor/imager [5]; Hsiao-Wen Zan et al. fabricated an a-IGZO visible-light photo-TFT by employing a polymeric light absorption capping layer [6]; and T. H. Chang et al. improved a-IGZO photo-TFTs by optimizing the oxygen partial pressures during the deposition, resulting in a high responsivity (*R*) over 4.75 A/W [7]. Compared to conventional two-terminal photoconductors, a-IGZO photo-TFTs usually have a much higher photosensitivity (P) by selecting the operation voltage appropriately, leading to enhanced sensitivity to the irradiation [8]. However, due to the relatively low visible transmittance of a-IGZO thin films, the introduction of a-IGZO photo-TFTs will result in the sacrifice of aperture ratio. To solve this problem, most researchers have attempted to increase the field-effect mobility (*μ_FE_*) of a-IGZO TFTs and thus reduce the switch size. L. X. Qian et al. treated the a-IGZO TFT with HaLaO gate dielectric in a CHF_3_/O_2_ plasma, and thus increased its *μ_FE_* to an ultrahigh value of 39.8 cm^2^/Vs [3]. Moreover, G. M. Wu et al. proposed a-IGZO TFTs with a *μ_FE_* of 33.5 cm^2^/Vs by using atmospheric pressure plasma treatment on e-beam deposited silicon dioxide gate dielectric layers [9]. Very recently, L.L. Zheng et al. fabricated a high-mobility a-IGZO TFT (*μ_FE_* > 60 cm^2^/Vs) with an atomic-layer-deposited SiO_2_ gate insulator [10]. Nevertheless, it is still quite difficult to realize the *μ_FE_* of a-IGZO TFTs over 70 cm^2^·v^−1^·s^−1^ so far. Recently, it was reported that the incorporation of light main-group metals—for example, Al, Mg and Ca—in oxide semiconductors could lead to improved optical transmission and enlarged bandgap (*E_g_*) [11]. For instance, ZnMgO (3.68 eV) presents a larger *E_g_* than ZnO (3.37 eV), and so can act like a barrier layer to build up ZnMgO/ZnO heterojunction and realize a high-electron-mobility transistor (HEMT) [12,13]. In this work, we tried to increase aperture ratio by enlarging the transmittance of photo-TFTs for each sensor pixel. Accordingly, the photo-TFT based on InGaMgO (IGMO) film, in which Zn was completely replaced by Mg, was firstly proposed, and its UV photodetection characteristics were comprehensively investigated. Moreover, the post-deposition annealing (PDA) in ambient oxygen was conducted to further optimize film quality and device performance. It was found that the density of oxygen vacancies (O_vac_) in IGMO film was effectively reduced with the PDA treatment, leading to the decrease of dark current (*I_dark_*) and the increase of photo current (*I_photo_*). As a result, the UV photodetection characteristics of IGMO photo-TFT—including *P*, R, and internal gain (*G*)—were dramatically improved.

## 2. Experimental Details

**Figure 1:**
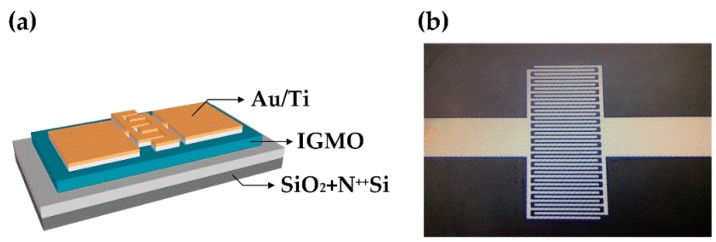
(**a**) Schematic and (**b**) optical microscope image of IGMO photo-TFT.

The schematic and typical optical microscope image of IGMO photo-TFTs are shown in Figure 1a,b, respectively. Firstly, n-type (100) heavy-doped Si substrates (0.01 Ω·cm) with a 100-nm thermal grown SiO_2_ dielectric film at the top were cleaned by the conventional Radio Corporation of America (RCA) method. Secondly, IGMO films were synthesized on each sample by plasma-assisted molecular beam epitaxy (MBE) at room temperature. In order to compare with a-IGZO (In: Ga:Zn = 2:2:1) fairly, the composition of a-IGMO film was maintained at In: Ga:Mg = 2:2:1 [14]. During the synthesis, the background vacuum in the growth chamber was about 3 × 10^−8^ torr, the RF power of O_2_ plasma was fixed at 300 W, and the O_2_ flow rate was fixed at 2 sccm by a mass flow controller. Thirdly, considering the ultra-high vacuum demanded in MBE chamber, the flow rate of O_2_ must be lower than 4 sccm, which is far from enough for the optimization of the oxygen contents in a-IGMO films, even with the assistance of RF plasma. Therefore, the O_2_ PDA treatments at 350 °C in the furnace were conducted at different time periods (0, 120, and 240 min). The PDA treatment was employed at the atmospheric pressure with a fixed O_2_ flow rate of 1.2 × 10^3^ sccm. Finally, the conventional lift-off process was utilized to form the interdigital source/drain electrodes, which consisted of 30-nm Ti and 50-nm Au deposited by e-beam evaporator. The width, length, and spacing of the electrode fingers were 12.5, 500, and 12.5 μm, respectively. 

As for the material properties, the crystallinities, thicknesses, transmittance spectra, and binding energies of the IGMO films were investigated by Bede D1 multi-functional X-ray diffraction (XRD), Veeco Dektak 150 surface profiler, ultraviolet spectrophotometer PERSEE TU-1810, and XSAM800 X-ray photoelectron spectroscopy (XPS), respectively. In addition, the current-voltage (I–V) characteristics of IGMO photo-TFTs were measured by an Agilent 4155B semiconductor parameter analyzer. Lighting emitting diode (LED) lamps with different wavelengths were employed as the light sources during the photosensitivity evaluation.

## 3. Results and Discussion

**Figure 2:**
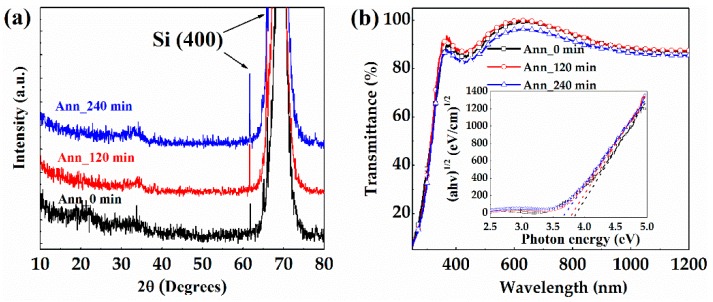
(**a**) XRD spectra of 0-, 120-, and 240-min annealed IGMO films; (**b**) transmittance spectra of a-IGMO films, the inset was the plots of (*αhν*)^1/2^ vs. *hν*.

Figure 2a shows the XRD spectra of the IGMO films with different PDA time. For each sample, there is no evident peak except the (400) diffraction peak of Si substrate [15], implying that all samples remain in the amorphous phase even after the PDA treatment. This can be attributed to the room temperature deposition and relatively low PDA temperature of 350 °C. Furthermore, the transmittance spectra of a-IGMO films grown on vitreous quartz substrate were measured as shown in Figure 2b. All the samples exhibited high transmittances of approximately 90% in visible spectra, clearly larger than the previously-reported a-IGZO films, whose visible spectral transmittances are approximately 80% [16,17,18]. This has demonstrated the possibility of our proposed a-IGMO photo-TFTs for enlarging the aperture ratio of the sensor pixel. Furthermore, the *E_g_* of a-IGMO films were extracted based on Tauc’s law as listed below [19]:

(1) $${\left(\alpha h\upsilon \right)}^{1/2}=A(h\upsilon -E_{g})$$

where *α* is the absorption coefficient, *hν* is the incident photon energy, and *A* is a constant. In general, *α* can be expressed by the Equation [20]:

(2) $$\alpha =\frac{1}{t}\ln\left(\frac{1}{T}\right)$$

where *t* is the thickness of a-IGMO film, and *T* is its transmittance. The thicknesses of the 0-, 120-, and 240-min annealed a-IGMO films were 54.2, 49.4, and 48.3 nm, respectively. As shown in the inset of Figure 1b, *E_g_*’s were determined to be 3.81, 3.78, and 3.62 eV for the 0-, 120-, and 240-min annealed a-IGMO films, respectively. Note that, all the values are larger than that of a-IGZO films (~3.1 eV) [21]. We attribute the monotonically decreasing *E_g_* of a-IGMO films with prolonging PDA time to the Burstein-Moss (BM) effect [22,23]. It was reported that *E_g_* can be enlarged with the increase of carrier concentration due to the occupation of the states near conduction band minimum (CBM) and accordingly the blue-shift of the optical absorption edge. According to XPS analysis, the percentage of oxygen atoms in the 0-, 120-, and 240-min annealed a-IGMO films are 41.3%, 45.4% and 52.7%, respectively (much lower than the stoichiometric ratio of 58.3%), suggesting our prepared a-IGMO films are all highly oxygen deficient. Usually, oxygen vacancies act as donor states in n-type metal oxide semiconductors and release electrons [24]. In our case, with the PDA treatment, the conduction band states near CBM in a-IGMO film become unoccupied due to the reduction of electron concentration, thus resulting in the narrowing of *E_g_*.

**Figure 3:**
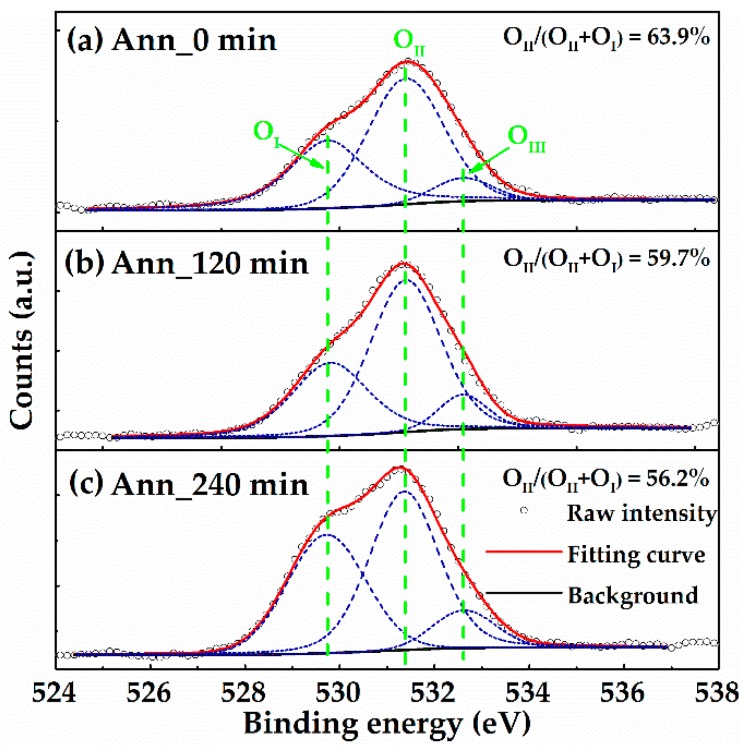
O 1 s XPS spectra of (**a**) 0-min; (**b**) 120-min; and (**c**) 240-min annealed a-IGMO films.

The electrical properties of a-IGMO films were extracted from the results of Hall-effect measurement, and listed in Table 1. It reveals that the electrical resistivity of a-IGMO films increase monotonically when prolonging the PDA periods, due to the decrease of both electron concentration (N_e_) and Hall mobility (*μ*_Hall_). It is attributed to the reduction of O_vac_ by PDA treatment in oxygen. Figure 3 exhibits the XPS spectra of O 1s core level for a-IGMO films with different PDA time. The binding energies have been calibrated according to the reference of C 1 s at 284.8 eV [25]. All the O 1s peaks were analyzed through Lorentzian-Gaussian fitting, and separated into those centered at (1) ~529.7 eV (O_I_), which represents the metal-oxygen bonds (M-O) without O_vac_; (2) ~531.4 eV (O_II_), which represents the M-O in oxygen deficient regions; and (3) ~532.6 eV (O_III_), which represents the loose oxygen bonds on the surface related to hydroxide (O-H) [26]. Accordingly, the atomic ratios of O_II_/(O_II_+O_I_) are 63.9%, 59.7%, and 56.2% for the 0-, 120-, and 240-min annealed a-IGMO films, respectively. Clearly, the as-deposited a-IGMO film has the highest density of O_vac_ within all the samples, and the O_vac_s were effectively suppressed with the prolonging of PDA time which further confirmed the results of Hall-effect measurement.

**Figure 4:**
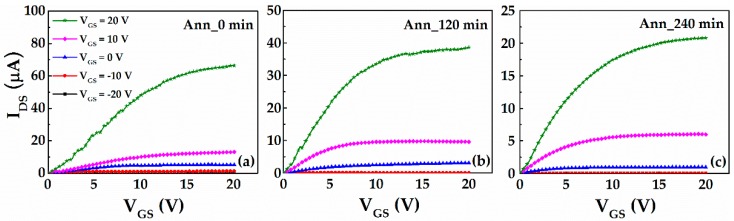
Output characteristics of (**a**) 0-min; (**b**) 120-min; and (**c**) 240-min annealed a-IGMO photo-TFTs.

**Figure 5:**
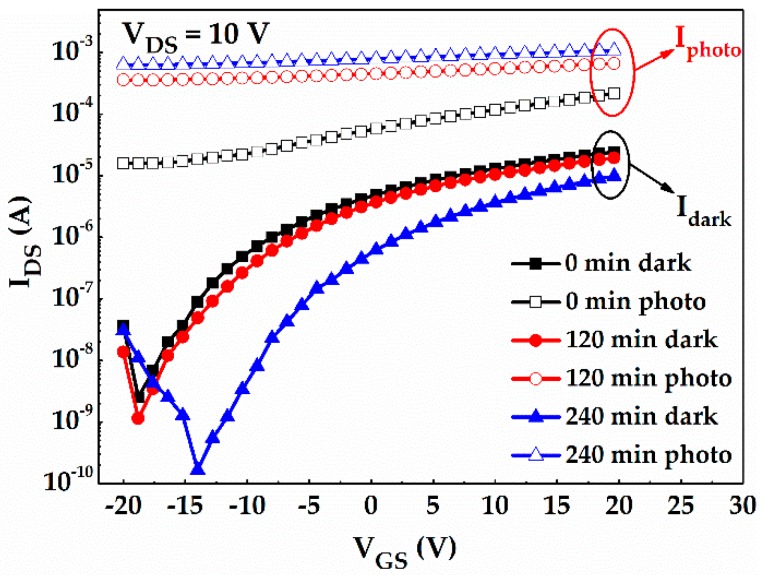
Transfer characteristics of a-IGMO photo-TFTs in dark and under 350-nm UV radiation.

**Figure 6:**
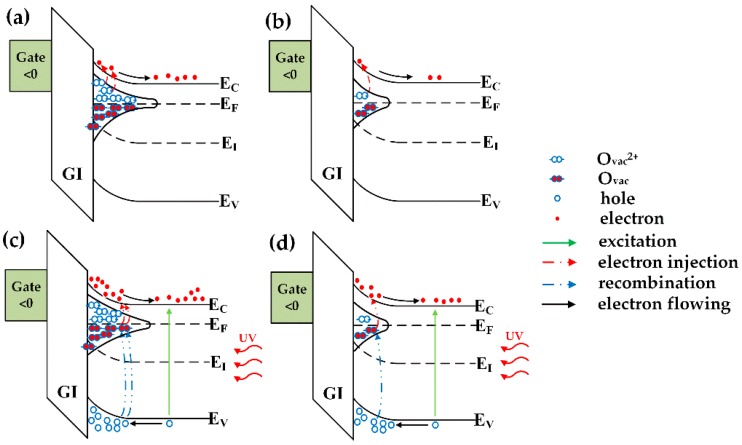
Energy band diagram of: (**a**) as-deposited a-IGMO photo-TFT in dark; (**b**) annealed a-IGMO photo-TFT in dark; (**c**) as-deposited a-IGMO photo-TFT under 350-nm UV illumination, and (**d**) annealed a-IGMO photo-TFT under 350-nm UV illumination. All the devices are in off states.

As shown in Figure 4, the output characteristics of a-IGMO photo-TFTs with different PDA time were measured in dark. All the devices exhibit typical n-type TFT output characteristics with distinct linear and saturation regions. Figure 5 shows the transfer characteristics of a-IGMO photo-TFTs, which were measured under a drain-to-source voltage (*V_DS_*) of 10 V in dark and under 350-nm radiation, respectively. It was found that *I_dark_* was decreased about one order of magnitude with prolonging the O_2_ annealing time from 0 to 240 min, as shown in Figure 5. It can be attributed to the reduction of O_vac_s, which act as shallow donor states (Figure 6a,b), as revealed by the previously discussed XPS result. Furthermore, the subthreshold swing (*SS*) and the trap density at/near the semiconductor/dielectric interface (*N_it_*), was determined according to the equations [27]:

(3) $$SS={\left[\frac{\partial \log\left(I_{DS}\right)}{\partial V_{GS}}\right]}^{-1}$$

(4) $$N_{it}=\left[\frac{SS\log\left(e\right)}{kT/q}-1\right]\frac{C_{OX}}{q}$$

where *k* is the Boltzmann constant, *T* is absolute temperature, *C_OX_* is the gate-oxide capacitance per unit area, and *q* is the electronic charge. Accordingly, the *SS* were 0.87, 0.69, and 0.48 V/dec, while the *N_it_* were calculated to be 8.01 × 10^11^, 6.24 × 10^11^, and 4.16 × 10^11^ cm^−3^ for 0-, 120-, and 240-min annealed a-IGMO photo-TFTs, respectively. It was revealed that both *SS* and *N_it_* decreases as the PDA time rises from 0 to 240 min, partly demonstrating the effect of PDA treatment in oxygen on the suppression of defects.

When the devices were illuminated by a 350-nm and 25-μW/cm^2^ UV light, *I_photo_* was significantly increased with the prolonging of the PDA time from 0 to 240 min. According to previous reports, *I_photo_* in photo-TFTs might be reduced by the decrease of O_vac_-related defects due to the suppression of photoconductive internal gain, which is contrary to our result [28]. In fact, O_vac_s can also form deep states in the forbidden gap, which often act as effective recombination centers of photogenerated carriers as illustrated in Figure 6c,d [29]. Hence, we suggest the observed increase of *I_photo_* should be ascribed to the reduction of recombination centers with the PDA treatment in oxygen. Note that, in the 120-min annealed sample, the suppression effect of O_2_ annealing on O_vac_s might be more obvious for the deep states due to their larger density. Consequently, it led to the different levels of impact on photo and dark currents. When the annealing time prolonged to 240 min, both deep and shallow O_vac_-related states were intensively reduced simultaneously, resulting in the significant change in both photo and dark currents. To further evaluate the photodetection properties of a-IGMO photo-TFTs, the *P*, *R*, and *G* were also determined according to the following equations [30]:

(5) $$P=\frac{signal}{noise}=\frac{I_{photo}}{I_{dark}}$$

(6) $$R=\frac{I_{photo}-I_{dark}}{P_{opt}}$$

(7) $$G=\frac{I_{photo}-I_{dark}}{q\eta }\left(\frac{h\nu }{P_{opt}}\right)$$

where *η* is the quantum efficiency, and *P_opt_* is the incident light power. Here, *η* is assumed to be equal to 1, which means that an incident photon generated one pair of electron and hole [31]. In order to compare their best photodetection characteristics, different V_GS_s, which correspond to the lowest dark current for each sample, were selected. Accordingly, *P’s* were determined to be 6.3 × 10^3^, 3.1 × 10^5^, and 3.9 × 10^6^ for 0-, 120-, and 240-min annealed samples, respectively. Clearly, *P* of a-IGMO photo-TFTs was significantly improved by prolonging the PDA time. Similarly, both *R* and *G* were increased with a longer PDA treatment time, and listed in Table 1. In addition, there are extremely high *Gs* in our proposed devices, which is attributed to the holes trapping by O_vac_-related defects. As a result, the 240-min annealed a-IGMO photo-TFTs exhibits the best device performance, including the lowest *I_dark_* of 1.7 × 10^−10^ A, the largest *P* of 3.9 × 10^6^, and the highest *R* of 1.5 × 10^4^ A/W. All the parameters are listed in Table 1 for comparison. However, the turn-on voltages (V_on_s) are too negative as shown in Figure 5, and the further optimization might be required in the future work, for example, by prolonging the O_2_ annealing time, increasing the O_2_ flow rate, or raising the annealing temperature.

**Figure 7:**
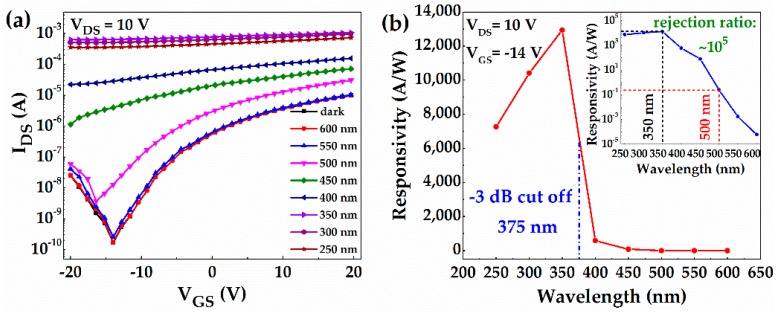
(**a**) Transfer characteristics vs. wavelength; (**b**) spectral response characteristics of the 240-min annealed a-IGMO photo-TFT.

Figure 7a reveals the *λ*-dependence of the 240-min annealed photo-TFT, which was measured by exposing them to 600-, 550-, 500-, 450-, 400-, 350-, 300-, and 250-nm lights with the fixed power of 25 μW/cm^2^. Under UV illumination, *I_DS_* increased significantly, which can be attributed to the generation of non-equilibrium carriers [32]. Moreover, the shorter the light wavelength, the more obvious the increase of *I_DS_*. In particular, the a-IGMO photo-TFT cannot be effectively modulated by *V_GS_* once upon the UV illuminations (*λ* < 400 nm). Moreover, threshold voltage (*V_TH_*), which was extracted from the equation [1]:

(8) $$I_{DS}^{1/2}=\sqrt{\frac{W}{2L}\mu_{FE}C_{OX}}(V_{GS}-V_{TH})$$

negatively shifted in the 240-min annealed a-IGMO photo-TFT with the reduction of illumination light wavelength, which further confirmed the increase of photogenerated carriers. In addition, the spectral response characteristics of the 240-min annealed a-IGMO photo-TFT at *V_GS_* of –14 V and *V_DS_* of 10 V is exhibited in Figure 7b, in which the inset shows the plot in logarithmic scales. It is clear that the a-IGMO photo-TFT is only sensitive to illumination with a *λ* less than 450 nm. The peak response occurs at ~350 nm, and the −3 dB cutoff wavelength is ~375 nm, which indicates our proposed device is a true UV photodetector. According to the determined bandgap (3.62 eV), the intrinsic-absorption wavelength can be calculated to be ~340 nm. In contrast, the −3 dB cutoff wavelength is generally larger than the intrinsic-absorption one due to the transition from tail-states and other sub-bandgap defect states to the conduction band [33]. Moreover, the UV-to-visible rejection ratio, which is defined as the ratio of *R* at 350 and 500 nm [34], is about five orders of magnitude, proving the high sensitivity of our device. The observed decrease of *R* when the *λ* is less than 350 nm might be attributed to the exciton-exciton annihilation—i.e., the interaction of excitons with each other when their density is dramatically enhanced by high-energy photons [30]. As seen in Table 2, the 240-min annealed a-IGMO photo-TFT is compared with various previously reported a-IGZO UV photodetectors. Clearly, our fabricated device exhibits an extremely high performance in the key parameters.

## 4. Conclusions

In conclusion, the photo-TFT based on a-IGMO thin film has been proposed and fabricated, and a-IGMO exhibits larger bandgap and higher visible transmittance in comparison to a-IGZO. Moreover, the XPS results reveal that the PDA treatment in oxygen can effectively suppress the density of O_vac_s in a-IGMO film, thus significantly reducing the *I_dark_*, *SS*, and *N_it_* of the photo-TFT. Furthermore, a dramatically increased *I_photo_* was observed, which may be attributed to the reduction of recombination centers related to O_vac_s. As a result, a continuous improvement in *P*, *R*, and *η* was exhibited by prolonging the PDA time from 0 to 240 min, and the 240-min annealed sample exhibited the lowest *I_dark_* of 1.7 × 10^−10^ A, the highest *P* of 3.9 × 10^6^, and the largest *R* of 1.5 × 10^4^ A/W to 350-nm UV radiation, exhibiting more outstanding performance compared to many previously-reported amorphous UV photodetectors. In summary, this work has demonstrated that a-IGMO photo-TFT is a promising alternative for UV photodetection, especially for application in transparent contact-free interactive displays.

## Figures and Tables

**Table 1:** Electric and UV photodetection characteristics of a-IGMO photo-TFTs with different PDA time.

PDA Time [min]	E*_g_* [eV]	*μ*_Hall_ [cm^2^/Vs]	N_e_ [cm^−3^]	Resistivity [Ωcm]	*I_dark_* [A]	*I_photo_* [A]	*P*	*R* [A/W]	*G*
0	3.81	2.6	7 × 10^15^	3.5 × 10^2^	5.2 × 10^−9^	1.6 × 10^−5^	6.3 × 10^3^	2.9 × 10^2^	1.0 × 10^3^
120	3.78	2.5	4.5 × 10^15^	5.4 × 10^2^	1.1 × 10^−9^	3.6 × 10^−4^	3.1 × 10^5^	6.5 × 10^3^	2.3 × 10^4^
240	3.62	2.1	2.7 × 10^15^	1.1 × 10^3^	1.7 × 10^−10^	6.5 × 10^−4^	3.9 × 10^6^	1.5 × 10^4^	4.2 × 10^4^

**Table 2:** Comparison of the device performance of the present a-IGMO UV photo-TFT and some reported a-IGZO UV photodetectors.

Thin Film Material	Device Type	*E_g_* [eV]	*P*	*R* [A/W]	Rejection Ratio	Ref.
a-IGMO	Photo-TFT	3.62	3.9 × 10^6^	1.5 × 10^4^	10^5^	this work
a-IGZO	Photo-TFT	3.0	NA	4.75	NA	[7]
a-IGZO	Photo-TFT	3.2	10^4^	NA	NA	[35]
a-IGZO	MSM	3.0	NA	4 × 10^−3^	NA	[36]
a-IGZO	MSM	3.18	10^2^	1 × 10^−4^	10^2^	[37]
